# Environmental quality shapes the fitness payoffs of multiple paternity

**DOI:** 10.1186/s12862-025-02478-5

**Published:** 2025-12-01

**Authors:** Fragkiskos Darmis, Anja Guenther

**Affiliations:** 1 RG Behavioural Ecology of Individual Differences, Max Planck Institute for Evolutionary Biology, 24306 Plön, Germany; 2 Department of Zoology and Animal Ecology, University of Hildesheim, 31141 Hildesheim, Germany

**Keywords:** Bayesian, Environment, Environmental quality, Fitness, Food quality, Mammals, Mice, Multiple paternity, Polyandry, Rodents

## Abstract

**Background:**

Polyandry is widespread in nature and, in polygynandrous species, can lead to multiple paternity when a litter is sired by more than one male. Such multiply-sired litters have been suggested to produce benefits in low-quality environments that may be masked in higher-quality environments. So far, however, the effect of environmental quality has only been tested in birds with equivocal evidence. Here, we use 202 female house mice (*Mus musculus domesticus*) from 5 generations (4 years; N_observations_=255) that lived in semi-natural enclosures. We provided different enclosures with a different food quality to test the environment dependency of multiply-sired litters as well as its fitness consequences for females.

**Results:**

As the operational sex ratio became male-skewed, the incidence of multiple paternity increased, indicating that under high male-male competition males might coerce females or females might mate multiply to reduce infanticide risk. We also found that the advantages of polyandry depended on environmental quality: only in poorer quality environments females that produced offspring with multiple males weaned significantly larger litters. Variation in lifetime reproductive success was significantly predicted by the female tendency towards multiple paternity, with this relationship showing a complex non-linear pattern in both environments. Importantly, our results suggested that polyandry provides greater lifetime fitness benefits when resources are of poorer quality. In other words, polyandry potentially yields its greatest advantages when resources are a limiting factor, but contributes little when conditions are already favourable.

**Conclusions:**

Our study shows that ecological and social conditions interact to shape the fitness consequences of polyandry, with resource quality emerging as a key factor. These context-dependent benefits highlight how resource availability may influence the evolutionary maintenance of polyandrous mating in multi-male, multi-female systems.

**Supplementary Information:**

The online version contains supplementary material available at 10.1186/s12862-025-02478-5.

## Introduction

Polyandry, when females mate with multiple males, is now recognized as a widespread [1–6] and potentially adaptive reproductive strategy [5–8], rather than simply a byproduct of male coercion [9] that only enhances male fitness [1–4, 10–13]. Indeed, across taxa, polyandry has been linked to increased reproductive success in both humans and non-human females [14–18].

Polyandry can lead to extra-pair paternity in socially monogamous systems [1, 4], and to multiple paternity in polygynandrous mating systems when litters are sired by more than one male [3, 19]. Some hypotheses that explain its adaptive value include indirect benefits [1, 4, 8, 20, 21], such as increased offspring genetic variability [22, 23], fertilization assurance [24] or good genes [7, 25]; and direct benefits such as reduced male harassment and infanticide [6, 19, 26] or increased material benefits to females [27]. For example, in house mice (*Mus musculus domesticus*), multiple paternity increases when males show small differences in scent-marking [28], potentially because females try to obscure paternity and reduce infanticide or because male quality is difficult to assess. Importantly, some studies in birds have revealed that female and male age interact and shape paternity share [29, 30], and that older females are more likely to have multiply-sired broods [31]. However, these results are not consistent across species [32].

Overall, the evidence remains inconsistent regarding the underlying causes and the adaptive value of females producing litters sired by multiple males [1, 33–35], even within the same species [22, 36]. These conflicting findings have prompted the suggestion that environmental conditions underlie the causes and benefits of polyandry, known as the “context-dependent hypothesis” [37–39]. Under this framework, high-quality environments may obscure genetic advantages [40] by reducing heritable variation in offspring fitness [39], hereby minimizing any benefits of mating with multiple males. Conversely, in low-quality environments, offspring of superior genetic quality (presumed to be those sired through extra-pair matings) should enjoy a relative fitness advantage. Some findings support this idea [41–43]. In common yellowthroats (*Geothlypis trichas*), extra-pair offspring show a stronger immune response in a cold (i.e., unfavourable) environment. However, other studies have provided a link between female polyandry and high-quality environments [37, 38]: in serins, higher food abundance predicts higher incidence of extra-paired offspring [38]. Consequently, experimental tests of the context-dependent hypothesis have yielded conflicting results, and importantly, have been conducted almost exclusively in birds [42, 44], that is in socially monogamous systems [4, 45, 46]. In other words, there is a need to evaluate both the prevalence of extra-pair matings, as well as the adaptive significance of polyandry, across a wider range of taxa to fully assess any fitness consequences. Lastly, the role of ecological variation in influencing polyandry, particularly variation in resource quality, rather than just resource quantity, remains largely unexplored and is likely to be of crucial importance to understanding the ecological drivers of female mating.

Here, we investigate if and how the resource environment (food quality) affects multiple paternity in a polyandrous mammal living in semi-natural enclosures, the house mouse (*Mus musculus domesticus*). Our approach in this study, i.e., monitoring every individual’s lifetime reproductive output in a population, is rarely achieved, particularly for mammals in natural or semi-natural settings [47], and combines subcutaneous RFID PIT tagging with robust fitness measures (details in Methods). House mice are ecological opportunists that thrive in variable habitats, such as barns [48], where food is abundant [49]. Females are known to produce multiply-sired litters, making them an excellent model for exploring the context-dependence of multiple paternity.

We first constructed semi-natural enclosures designed to mimic the environment of wild populations. Mice in these enclosures developed densities and population structures similar to those observed in natural systems [49], allowing us to monitor the entire population longitudinally. We then manipulated environmental conditions by varying the quality of food provided in each enclosure, thereby altering the resource environment. Food was offered *ad libitum*, using either a standard- or high-quality pellet diet (see “Methods” for details, but note that standard-quality food is of lower nutritional quality than high-quality food), to simulate conditions typical of feral populations where food availability is generally abundant [48, 49]. Importantly, the quality of the food affects generation time, fecundity rate, stress-related behavioural and physiological responses in populations of mice [50, 51], and mice in standard-quality food consume more food on a daily basis [50]. Therefore, we expected female reproductive behaviour (i.e., multiple paternity and its fitness consequences) to vary as a function of the resource quality, that is across semi-natural enclosures.

We applied the predictions from the bird-literature, that polyandry might provide more benefits in low-quality environments, to our mammalian system. Specifically, we assumed that the potential drivers and fitness benefits of multiple paternity in mice are context-dependent, i.e., more pronounced in standard-quality enclosures (Table 1). We first tested whether the incidence of multiple paternity depended on the resource environment. Then, drawing on variables well-established in the literature as predictors of mating decisions and of multiply-sired litters (via extra-pair or multiple paternity), we formulated and tested three hypotheses. In each case, we explicitly considered the role of food quality.

Our first hypothesis (H1) was that the likelihood of multiple paternity increases with female age, as found in birds [29, 30], but that this link is context-dependent. We predicted (P1) that this effect would be stronger in standard-quality environments because older females [ranged in our data from 1 to 9 months, so they represent the upper end of the reproductive lifespan in this species: 52, 53] may compensate for reduced investment in offspring quality by increasing offspring genetic diversity through multiple mating, or because males become less effective at preventing cuckoldry as females age and possibly become stronger. Specifically, we expected older females on a standard-quality diet to show higher rates of multiple paternity than those on a high-quality diet, since standard-quality females face longer generation times [50] but more limited reproductive energy. Our second hypothesis was that multiple paternity increases with the strength of sexual selection, and that this effect would also depend on the environment (H2). We predicted that as the operational sex ratio, a measure of reproductive competition [54], becomes more male-biased male–male competition and male variance in mating success would increase, leading to higher rates of multiple paternity, consistent with the “breeding density hypothesis” [55]. This may result through sexual conflict: males may coerce females or commit infanticide [common in mice: 28] when mating competition is high. Consequently, females may mate multiply to avoid the costs of coercion or infanticide (“convenience polyandry” and “infanticide avoidance”, respectively). We expected this effect to be stronger in standard-quality food, where limited resources constrain female reproduction (P2). Finally, we hypothesized (H3a & H3b) that the fitness benefits of multiple paternity differ between resource environments, as suggested in avian systems [39, 40, 42]. In the short term, we predicted females with multiply-sired litters to wean larger litters [22, 56, 57], either through increased genetic variation among offspring [37, 58] or reduced infanticide risk [26]. In the long term, we predicted that these females would achieve higher lifetime reproductive success compared to those with singly-sired litters. Importantly, to test this, we measured all litters (singly- and multiply-sired) produced by each female throughout her whole lifespan in the enclosure and calculated fitness as the total number of pups produced. In both cases, we expected benefits to be visible only in standard-quality enclosures because high-quality resources may mask them.

## Materials and methods

### Animals

Female mice (*N* = 1110) lived in four semi-natural enclosures (19.6 m^2^ each) across five generations [50]. Enclosures were covered by a roof, inaccessible to predators and designed to resemble the natural conditions under which house mice would establish a colony, like a barn [49, 59]. Those mice originated from wild populations sampled in the Cologne/Bonn region of Germany (CB *N* = 18 original breeding pairs; 50°45′N–51°N, 6°45′E–7°E). Descendants of those wild-caught mice, the founders of our semi-natural populations (*N* = 160, 80 males, 80 females), were distributed to replicate seminatural enclosures, each containing 20 males and 20 females. After releasing the founders, we monitored population developments every 4–5 weeks (i.e., conducted full population monitoring sessions). During these monthly monitorings, we caught all mice within an enclosure to measure their body mass, took a tissue (ear clip; DNA) sample of new individuals (weighing >10 g) to assign maternity and paternity using microsatellite analysis, and implanted new animals with an RFID PIT tag (Planet ID, 1.4 × 9 mm) for permanent recognition. All handling and sampling were performed on site and no local or general anaesthesia was used during ear clipping. This procedure is standard in small mammal field studies and was approved by the relevant animal ethics committee (see Ethics section). Following sampling, individuals were placed in standard cages until all animals had been processed, after which they were released back into their respective enclosures.

Whenever a given population reached at least 80 chipped offspring (corresponding to approximately 160 individuals in total), we removed (i.e., did not release back in the enclosure as stated above) the older generation to maintain population size below the estimated carrying capacity. Removed individuals were returned to laboratory housing for potential further study or ethical management, in full compliance with animal welfare regulations. This removal typically occurred 8 months (range 6–10) after the birth of the first generation and involved all individuals from the preceding generation that had completed most of their reproductive period. Across the study, population sizes per room varied between 39 and 200 individuals, with mean sizes ranging from 96 to 99 and medians between 86 and 91 individuals. Peaks above the ~ 160-individual threshold occurred intermittently, triggering removal events and producing cyclical fluctuations in population size. Populations in standard-quality food enclosures exceeded the removal threshold in 8% of monitoring dates and those in high-quality enclosures in 8.2% of dates, maintaining consistency across food quality treatments. Because of these removals, observations were restricted to a maximum female age of nine months, which nonetheless represents the upper end of the reproductive lifespan in this species [52, 53]. Within this time frame, individuals are able to complete the majority of their reproductive output.

Parentage was determined with 17 microsatellite markers using the procedure adapted from *Linnenbrink et al.* [60]. DNA extracted from the ear clips was amplified using Multiplex PCR kits (QIAGEN) and the samples were run on an ABI 3730 Sequencer (Applied Biosystems). GeneMarker (V2.6.4) was used to call alleles and Colony version 2.0.7.2 [61] to assign the parentages based on the maximum likelihood of each potential parental pair. After identifying the maternal identity of each pup via microsatellite parentage analyses, we determined litter size for each reproductive event by linking genetic data with monitoring records. For each mother, we compiled all her assigned offspring and identified the first date each pup was captured in the enclosure. Because pups are chipped at ~ 10 g (corresponding to an age of ~ 1 month), the date of first capture allowed us to group pups into litters. For example, if a female had four pups of the same age first captured during one monitoring session, and two additional pups of a similar (but different to the four above) age captured during a later session, we assigned these to two separate litters. Multiple paternity was recorded when pups from the same litter were sired by different males according to the parentage results (e.g., a litter of five pups with three sired by male A and two by male B was classified as multiply sired). Importantly, our estimates of litter size are based on the number of pups surviving to be chipped (≈ 10 g), and thus reflect litter size at weaning rather than at birth.

## Experimental design

We excluded observations of females that never reproduced and focused on all females that had at least one litter with more than 1 pup surviving to be chipped and genotyped (i.e., reaching at least 10 g in one of the monitorings). Using these cutoffs, we focused on 255 reproductive events (i.e., litters) from 202 females that lived in the seminatural enclosures across five generations. Two of the four enclosures (enclosures 1 and 2) always received standard-quality food Altromin 1324 (SQ) and the two others (enclosures 3 and 4) always high-quality food Altromin 1414 (HQ). Previous research has indicated the importance of the food quality and diet for house mice reproduction and behaviour [48, 51]. The standard-quality food contained 3227 kcal/ kg metabolizable energy (24% from protein, 11% from fat and 65% from carbohydrates) while the high-quality food contained 3680 kcal/kg (28% from protein, 22% from fat and 50% from carbohydrates). Of the 202 females, 101 were housed in enclosures 1 and 2, which both received standard-quality food, and 101 in enclosures 3 and 4, which both received high-quality food. For analyses, we grouped enclosures 1 and 2 together as the standard-quality food, and enclosures 3 and 4 together as the high-quality food. We combined observations of enclosures with the same food quality to increase our sample size and statistical power. This approach is justified because the only systematic difference between the enclosures is food quality. Specifically, within each enclosure, food and water are equally distributed across nine stations and are replenished daily to be *ad libitum*, to mimic natural house mice populations [in barns or fields: [49, 62]. Each enclosure had natural daylight and temperature fluctuations, with underfloor heating keeping a minimum of 10 °C since mice were not able to dig burrows to escape cold temperatures. Each enclosure was littered with Aspen bedding and thirteen shelters provided nesting space. These conditions are similar to natural house mouse populations found in Europe where mice live commensally with humans [59].

## Variables measured

We quantified multiple paternity in each population as the proportion of litters sired by more than one male. For all litters with more than one pup, we recorded: (1) whether the litter was multiply-sired (yes/no), and (2) its size (number of pups). Additionally, we measured (3) the population size and (4) operational sex ratio, OSR [63], from the monitoring session closest to—but before—the estimated conception date (see section “Animals” for details on the monitoring procedure). The OSR was calculated as the ratio of sexually active males ≥ 2 months of age [as they rarely reproduce earlier: 64] divided by the number of females that were not highly pregnant or lactating. Last, we quantified (5) individual fitness as the total number of weaned offspring each female had over its lifetime and (6) female age per reproductive event (measured in months).

## Statistical analyses

R 4.4.1 (2024-06-14) was used in all analyses (R Core Team, 2024).

Focusing on offspring that reached at least 10 g, the minimum size at which pups can be successfully implanted with an RFID tag for paternity assignment, we first compared if there was a difference in the mean litter size and number of litters of females fed standard- *versus* high-quality food, using two Mann-Whitney U-tests.

To test whether social and intrinsic factors influence multiple paternity (H1 & H2; Table 1), we fitted one Bayesian generalized linear model [model 1; *brms* package 55] with whether each litter was multiply-sired (yes/no) as a binomial response and female ID as a random effect to account for repeated individual measures. To test H1, that older females are more likely to produce multiply-sired litters, we included female age at litter parturition and food quality as fixed effects, along with their interaction to test if this effect is stronger under a standard-quality food treatment. To test H2, that mating competition affects multiple paternity, we included the operational sex ratio (standardized, i.e., mean = 0, sd = 1) and food quality as fixed effects, as well as their interaction. We also included litter size and population size (standardized, i.e., mean = 0, sd = 1) as fixed effects, each interacting with food quality, since both influence the incidence of multiple paternity; and population size can further affect competition and the operational sex ratio [54, 63].

To explore if producing a litter with multiple sires has short-term or long-term benefits for females, and if any benefits are context-dependent [as suggested by seminal studies: 37,38,40,41], i.e., hypotheses 3a and 3b (H3a & H3b), we used three Bayesian models (models 2–4). To test H3a, if multiple paternity predicts a larger litter size only within standard-quality environments, we used litter size as a response variable regressed against the interaction between food quality with whether the litter was multiply sired (yes/no). We assumed a Poisson error structure, included age as a covariate and female ID as a random effect (model 2). After running model 2, we used a *post-hoc* test (*emmeans* package) to compare the predicted mean litter size of females with *versus* without multiple paternity, both between and within food quality treatments.

To test whether the long-term fitness effects of engaging in multiple paternity are context-dependent (H3b), we fitted one additional Bayesian generalized linear mixed-effects model for each food quality treatment. In both models, the response variable was whether a litter was multiply sired (yes/no, binomial error structure). Fixed effects included litter size, standardized operational sex ratio (OSR) and standardized population size. Female ID was included as a random effect to account for repeated measures within individuals. We did not include age as a predictor because our first model (model 1) showed it was not significantly correlated with the likelihood of multiple paternity. From each model, we extracted the best linear unbiased predictions (BLUPs) of each individual female and used them as predictors in two follow-up models (models 3&4) to examine their relationship with lifetime fitness across environments. BLUPs showed the random deviations of each female from the population average (tendency) to engage in multiple paternity, adjusted for OSR, population and litter size. The models testing H3b (models 3&4) were two generalised additive models (GAMs; using *brms* with a smooth command), one for each food quality. In both models, the response variable was female lifetime reproductive output (i.e., total number of offspring; assuming Poisson errors) and the main predictor was the individual BLUPs extracted from the corresponding food quality-specific model described above. We opted for GAMs because visual (Supp. Fig.S1) and statistical (using the WAIC; Supp. table S4) inspection indicated that the relationship between the BLUPs and fitness was non-linear and better captured by a smooth function. One advantage of using GAMs is their flexibility, as they can automatically adjust the degree of smoothness of the curve that best fits the non-linear relationship.

After fitting models 3 and 4, we visualized the effect of BLUPs on fitness in two complementary ways. First, we plotted the non-linear estimated effect from the GAMs (Fig. 3a). Second, to provide a more intuitive visualization of variation among individuals, we generated violin plots based on three quantile groups of BLUPs (below the 1st quantile, between the 1st and 2nd quantiles, and above the 2nd quantile) for each food quality treatment (Fig. 3b). This dual visualization highlights both the continuous non-linear relationship and the distribution of fitness outcomes across distinct subgroups of females.

Validity of the Bayesian generalised models was assessed visually (caterpillar plots of the posterior distribution), statistically using the Gelman-Rubin diagnostic [66] and by calculating the variance inflation factor (no predictors were highly correlated). The models assumed an uninformative prior, were run for at least 10,000 iterations (depending on convergence issues) for 4 chains and had a burn-in of 1000.

## Results

**Table 1:** Hypotheses, predictions and support for the hypotheses found in this study (+ supported; — not supported)

Hypotheses	Predictions	Support here	
**H1**: Among birds, older females often show more multiple paternity [29, 30] due to reduced investment in offspring or reduced male control of females. Since SQ^1^ females have longer generation times [50], this effect is stronger in SQ^1^.	**P1**: Older females show higher multiple paternity rates, and this is stronger for SQ^1^ females compared to HQ^1^ ones.	Model 1: —	
**H2**: The social (OSR) and resource (food-quality^1^) environment interact in shaping multiple paternity. Under male-skewed OSRs: males may coerce females more; or increase infanticide attempts due to heightened mating competition [67]; or females have more males available to mate.	**P2**: Multiple paternity increases as OSRs become male-biased and this effect is larger in SQ^1^ because mating opportunities are more costly than in HQ^1^ (individuals have more energy to allocate into reproduction in HQ^1^).	Model 1: —	
**H3**: Worse environments, here SQ^1^, yield higher fitness benefits of multiple paternity [39, 40, 42, 43]. We further hypothesised this effect to extend to lifetime reproductive success (H3b).	**P3**: Multiply-sired litters are larger in SQ^1^ and the lifetime fitness benefits of engaging in multiple paternity are evident for SQ^1^ females. In contrast, HQ^1^ conditions may mask the fitness effects of multiple paternity.	Models 2–4: +	

^1^Refers to the food quality (FQ) females received (standard/SQ or high/HQ).

On average, females receiving high-quality food weaned significantly larger litters (Mann-Whitney U-test: W = 4395.5, *p* = 0.031; standard-quality food = 3.3 ± 1.56 & high-quality food = 3.7 ± 1.56) but had a similar average number of litters compared to those receiving standard-quality food (Mann-Whitney U-test: W = 5624.5, *p* > 0.05; standard-quality food = 1.57 ± 0.65 & high-quality food = 1.52 ± 0.71). Overall, 96/255 litters in both food qualities were multiply-sired (~ 38%), 52/128 (~ 35%) in standard and 44/127 (~ 40%) in high-quality. About 32% of standard (40/127) and 38% of high-quality (49/128) food females produced at least one multiply-sired litter. Last, female age at litter conception ranged from 1 to 9 months, with a mean of 6.1 ± 1.1 months in the standard and of 5.6 ± 1 months in high-quality populations, respectively.

We found no support for hypothesis 1 (Supp. table S1) since older females did not show higher multiple paternity rates (est. = 0.25, CI = -0.36–1.27) and there was no interaction between age and food quality (est. = 0.19, CI = -0.64–1.10; Supp. table S1). Surprisingly, the likelihood of multiple paternity did not increase with larger litters (high-quality food: est. = 0.18, CI = -0.12–0.72; standard-quality food: est. = 0.25, CI = -0.22–0.83) and did not differ between food qualities either (est. = -2.14, CI = -6.78–1.25).

We found no support for hypothesis 2, i.e., an interaction between the OSR (high-quality food: range = 0.26–1.6, mean = 0.7; standard-quality food: range = 0.19–2.3; mean = 0.63) and the resource environment (est. = -0.62, CI = -1.82–0.34; Supp. table S1). Crucially though, the OSR was positively correlated with the probability of a litter to be multiply-sired in the high-quality environment (est. = 0.69, CI = 0.02–1.87; Fig. 2b; Supp. table S1). In contrast, in standard-quality conditions, the effect of OSR remained weak and did not reach statistical significance. Finally, a similar pattern was observed for population size (est. = 0.83, CI = 0.03–2.32; Supp. table S1), which significantly predicted a higher likelihood of multiple paternity only under high-quality conditions.

**Fig. 1:**
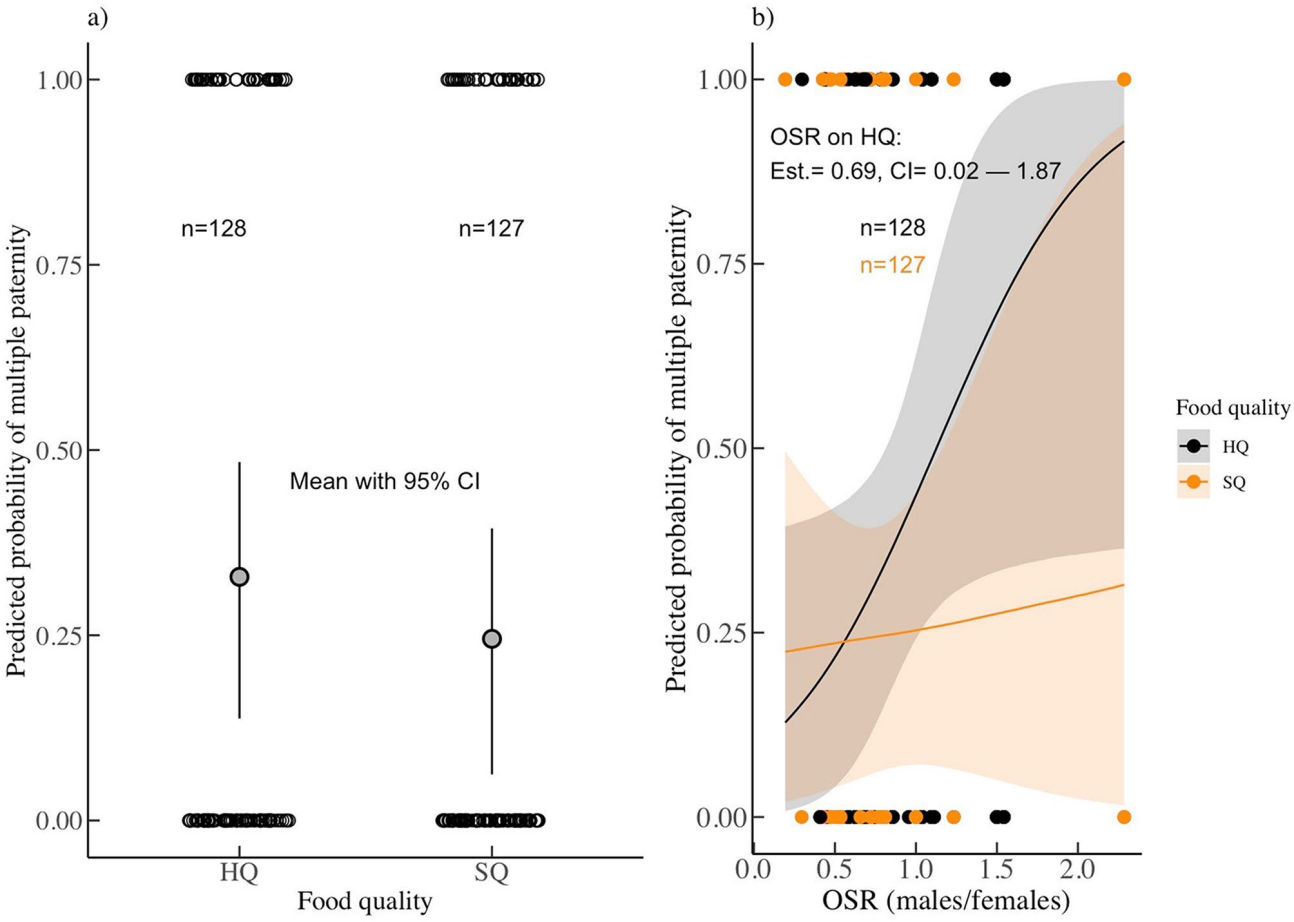
**a**: The mean probability (circle) of a female showing multiple paternity (y-axis) as a function of the environment (HQ = high-quality food; SQ = standard-quality food: x-axis) with 95% credible intervals; **b**: The probability of a female showing multiple paternity (y-axis) as a function of the operational sex ratio (number of adult males/reproductively active females; x-axis) across environments. The line indicates the predicted effect by varying the respective focal variable and the shaded areas the 95% credible intervals. Raw data are plotted and are jittered in both plots. On top, the effect size and the credible interval of the predictor variable on the response (y-axis) are shown. Sample sizes are given on top (n)

**Fig. 2:**
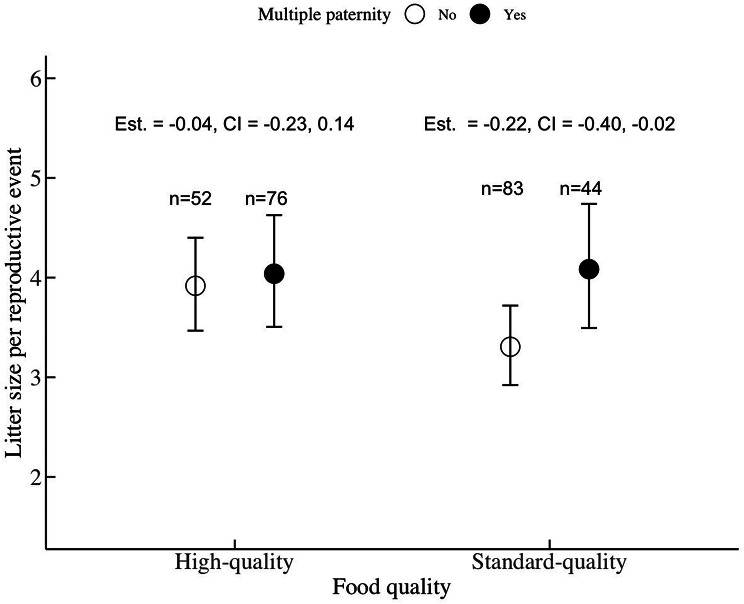
The mean (dot) litter size with (filled circle) and without (empty circle) multiple paternity under different food qualities (SQ = standard quality food; HQ = high-quality food). Only in standard quality food multiple paternity was linked to significantly larger litters at weaning. The lines indicate the 95% (lower and upper highest posterior density; HPD) credible interval. On top, the estimate and the credible interval for a litter of females without multiple paternity compared to females with multiple paternity within each food quality are shown. Sample sizes are given on top (n)

We found strong support for hypothesis 3a, that multiple paternity increased (short-term) fitness in a context-dependent way: multiply-sired litters were larger than single-sired ones only in the standard-quality environment (Fig. 2; Supp. table S3). Moreover, litter sizes were significantly reduced in females fed standard-quality food relative to those on a high-quality diet (est. = -0.22, 95% CI: -0.40 – -0.04).

**Fig. 3:**
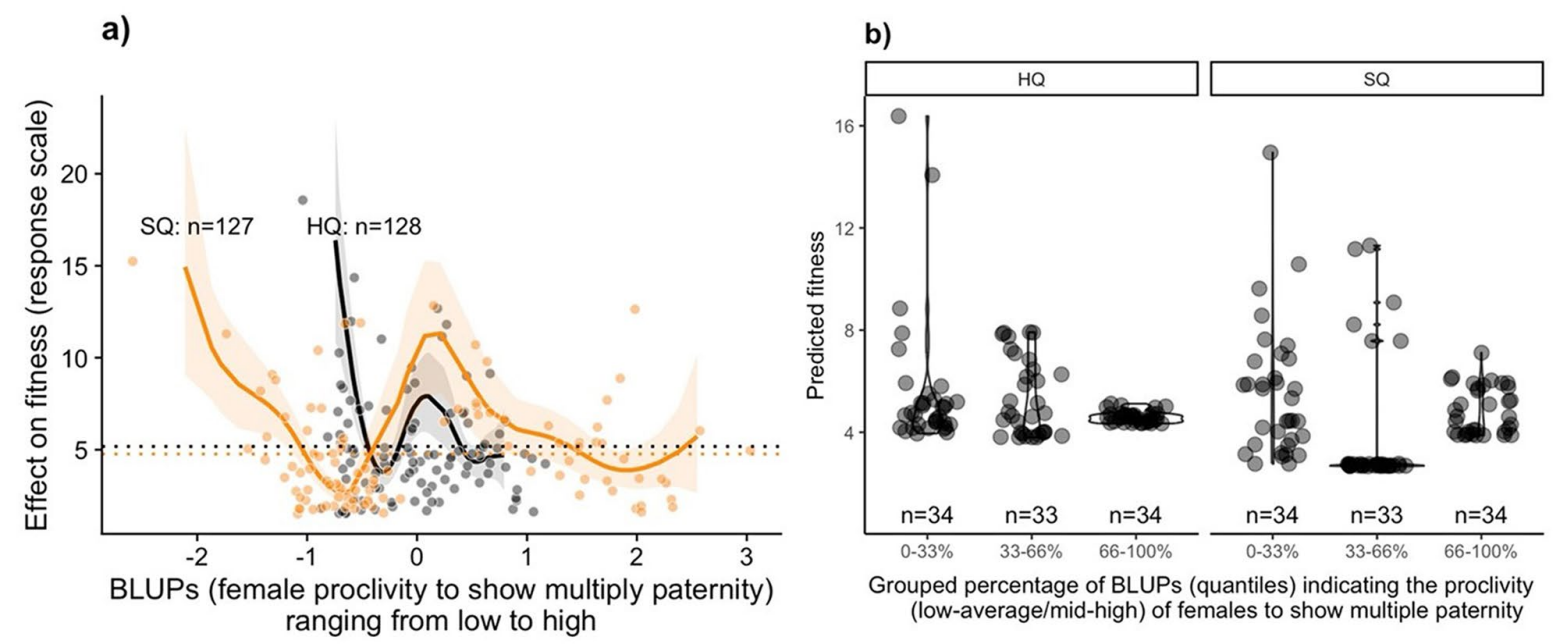
(**a**) Modeled effect of BLUPs (best linear unbiased predictors, representing individual propensity for multiple paternity relative to the population mean) on predicted fitness (response scale) under high-quality (HQ, solid black line) and standard-quality (SQ, dashed dark orange line) food. Shaded ribbons indicate 95% credible intervals; dotted horizontal lines show the mean fitness for each treatment. If the CI crosses the horizontal line, the effect is not different from the average BLUP (0). (**b**) Predicted fitness grouped by BLUP quantiles (low, mid, high) per food quality. Violin plots show the density of predictions, with jittered points indicating individual values. Plot (**a**) shows the continuous relationship, while plot (**b**) summarizes variation among individuals. Both panels highlight greater variation and a stronger BLUP–fitness relationship in the standard- compared to the high-quality food. Sample sizes are indicated (n)

We predicted the association between fitness and the propensity towards multiple paternity to be stronger in the standard-quality environment (H3b; models 3 & 4). While we could not directly assess any difference between food qualities and the BLUPs via an interaction term as in linear models, Fig. 3 demonstrates the effect of BLUPs within each food quality: (1) the propensity towards multiple paternity has greater implications in the standard-quality environment; (2) the variance in the propensity to mate multiply (BPUPs) was greater in the standard (range = -2.07–2.5) than in the high-quality (range = -0.73–0.78) environment (also see supp. Figure 3). Importantly, in the standard-quality food, the estimated among-female variance in the probability of multiple paternity was 3.96 (95% CI = 0.04–22.95), indicating substantial individual differences. In contrast, under high-quality conditions, among-female variance was considerably lower at 0.86 (95% CI = 0.002–6.85), suggesting that individual consistency was reduced when food was abundant; and (3) the propensity towards a high or low rate of multiple paternity (i.e., not at the population average) provides more benefits in standard-quality conditions, compared to high-quality (Fig. 3).

Importantly, in the standard-quality environment, females with below-average rates of multiple paternity showed fitness payoffs higher than the population average (Fig. 3). In both food treatments, the relationship between fitness and the tendency to engage in multiple paternity (as captured by BLUPs) was non-linear and complex, with effective degrees of freedom > 6. This indicates that the effect of BLUPs on fitness was strong but varied across the range of BLUP values. The GAM explained ~ 38% of the variation in fitness under high- quality conditions and ~ 61% under standard-quality conditions.

## Discussion

In this study, approximately one-third of litters (in both food qualities) were multiply-sired, in accordance with rates reported in other house mice [23%: 19; 29%: 34], rodent [68–70] and mammalian studies [34]. We found strong evidence for an interaction between environmental quality with mating competition and density effects in predicting the probability of multiple paternity. In contrast, age and litter size did not predict how many fathers a litter had, and the likelihood of multiple paternity was not lower for standard-quality females (i.e., found on a standard-quality diet) despite that they have smaller litters [50]. In other words, litter size alone was not statistically linked to multiple paternity tendency, although this correlation was positive (as predicted), which is in accordance with other reviews and meta-analyses across species [34, 35]. Importantly, while that litter size did not predict the likelihood of multiple paternity (model 1), the reverse relationship did occur (model 2): multiply-sired litters at weaning were larger under standard-quality conditions, an asymmetry that arose because the two models test different directions of causality (see below for detailed explanations).

In contrast to H1, age was not linked to the likelihood of multiple paternity and there was no interaction with food quality. Comparatively, existing studies show no consistent pattern when exploring the correlation between female age or experience with mating behaviour. For example, our results match a study in a lek mating system which found no link between multiple paternity and age [71]. Hence, we found support for the idea that female age (and consequently mating experience) might not directly affect female choice in mice [72]. However, in birds, that is in socially monogamous systems [29, 30, 73], age is a strong predictor of extra-group paternity and of the number of mates [69]. Consequently, maybe the link between age with polyandry and its consequences is tied to the mating system. Alternatively, because females produced fewer than two multiply-sired litters on average, the statistical power to detect age-related effects was likely limited, and this result should be interpreted with caution. Last, a possibility we did not test here is that mating decisions are shaped by the interaction between male and female age and/or experience, such that females may mate assortatively based on age, as observed in boobies [30]. For example, older female mice might associate only with males of specific age.

Surprisingly, litter size alone (i.e., number of pups in a litter) did not predict the incidence of multiple paternity despite that larger litters either provide more statistical power for multiple paternity to be detected, all other things being equal, or increase the potential fitness gain for males, which in turn might enhance male-male competition, coercion and multiple paternity [34, 35]. Consistent with our results, previous reviews across 49 mammalian species [34] and across 60 mammalian populations [35] reported a positive but statistically not significant correlation between the proportion of multiply-sired litters with average brood size and the mean number of sires, respectively. These studies relied on the average number of mates and mean litter sizes across species, therefore giving equal weight to each species regardless of the number of reproductive events, and here we confirm these findings using detailed reproductive data with multiple measurements per female. This approach allowed us to account for individual variation and potential intrinsic differences among females [21].

Under H2, we predicted that the operational sex ratio should be positively correlated with the likelihood of multiple paternity because under high male-male competition females either decrease infanticide and coercion by mating multiply or males coerce females. We further hypothesized that this effect would be stronger in the standard-quality environments, where resource quality can constrain reproduction (e.g., the rate of reproduction, litter size or the quality of offspring). However, our analyses revealed the opposite effect. Specifically, the incidence of multiple paternity increased as the operational sex ratio became male-biased in high-quality environments, as well as with increasing population sizes, revealing a context-dependent effect. Importantly, while similar positive relationships were detected for both population size and OSR in the standard-quality environment, they did not reach statistical significance (but note the large predicted credible intervals in standard-quality for the OSR: Fig. 2). Our results, nevertheless, align well with a recent comparative analysis, across reptiles, that found an increased incidence of multiple paternity as the adult sex ratio became male-skewed [74]. Our findings also align with previous work where higher population densities were associated with higher multiple paternity rates [19].

We propose several, non-mutually exclusive, explanations for why multiple paternity is positively linked to a male-biased OSR (and by extension to increasing population densities). First, under intensified male–male competition, males may resort to coercion to maximize reproductive success when access to females is limited [54]. Second, females may actively increase mating rates to reduce the risk of infanticide by confusing paternity. Third, multiple paternity may reflect increased female mating opportunities simply due to a greater number of available males. It may also be that because growth rates are faster in high-quality conditions [50], males attain a competitive size/threshold earlier than usual. In turn, this enables more males to successfully secure mates, either by attracting females (via female choice) or by coercing them. It is also possible that females in high-quality food environments rely less on territorial male support—such as protection against infanticide—which may be reduced when they mate with multiple males. This could allow them to incur the potential costs of multiple mating. While we have no direct evidence for such male care, recent work shows behavioural differences in the time males spend within territories [64], where females aggregate, suggesting that male behaviour could influence offspring survival in ways relevant to our findings.

Importantly, since both litter sizes and female breeding decline at higher densities [75, 76], likely due to negative effects of overpopulation on offspring fitness, most “stolen” paternity probably comes from males unable to monopolize access to females and exclude the others that actively seek extra matings. In other words, at higher densities, opportunities for multiple paternity per litter decrease because litter sizes shrink, yet overall multiple paternity rates remain elevated due to intensified mating competition. Focusing on male behaviour too, for example by analyzing the relative abundance of male alternative reproductive tactics—such as territorial and roamer males which have been only recently described in male house mice [64]—might be suitable to reveal how much these aforementioned patterns are driven by male behaviour [77]. It might be that as the number of adult males increases, more non-territorial males enter the mating pool (assuming a limited number of territories) and the costs of territoriality increase due to heightened male-male competition [78, 79]. In turn, females mate multiply to reduce infanticide risk or coercion from roamers which are less likely to ever reproduce [64]. Alternatively, females might simply seek genetic benefits [22]. Indeed, pups found in multiply-sired litters survive better to weaning [22]. Overall, while we cannot exclude that multiple paternity reduces infanticide risk in house mice [6, 19, 26], other social factors than the OSR must be studied to reveal a causal relation [4], with a focus on those social factors (e.g., reproductive tactics) that have a strong link to each species’ mating system.

We found support for H3, that multiple paternity increased (short-term) fitness (i.e., litter size at weaning) in a context-dependent way (Fig. 2): multiply-fathered litters were larger, compared to single-fathered litters, in the standard-quality environment only. This result is consistent with the idea that multiple paternity might offer context-dependent indirect genetic benefits. Our results build on previous studies in rodents [80], including house mice [22], where multiple paternity was linked to larger litters [but see contrasting results: 19, 36] by showing that this effect may be environment-specific; and that the fitness benefits of female polyandry may be as well (Fig. 3). Specifically, while larger litters at weaning did not predict a higher likelihood of multiple paternity (Fig. 1a), multiply-sired litters were predicted to be larger at weaning in the standard-quality, but not in the high-quality, environment (Fig. 2). Our results are consistent with both theoretical [81] and empirical studies [42, 44], which highlighted the context-dependency of female multiple mating [but those studies focused on birds and, therefore, extra-pair paternity: 42, 82, 83]. Here, we extend those findings to a mammalian polyandrous mating system involving multiple paternity. Notably, variance in litter size was greater in standard-quality environments, likely because the more nutrient-dense food in high-quality conditions enables females to wean larger litters regardless of whether they mate multiply.

The context-dependent effect of polyandry we observed may reflect increased offspring production and/or postnatal survival [22, 26]. These may result from enhanced offspring viability [58, 84], for example due to sperm competition, or more males (i.e., fathers) supplying resources to females [27], which in mice may involve access to food or to territories to give birth [48, 64]. Alternatively, they may result from reduced infanticidal attacks, something observed in other rodents too [26]. The latter seems plausible given that our results supported H2. In contrast, in high-quality resource environments—where individuals obtain more energy from comparable food intake—multiple paternity was not associated with larger litters at weaning. This is not surprising as females in such environments may have sufficient resources to invest in both self-maintenance and reproduction, thereby reducing life-history trade-offs. Consequently, the potential benefits of polyandry, such as weaning larger litters, are diminished, and females can achieve high reproductive output without necessarily mating multiply.

Similar to litter sizes, only in the standard-quality environments did multiple paternity rates deviating from the population average (as captured by BLUPs) translate into considerably higher fitness. In other words, for females in high-quality environments, BLUPs had little impact on predicted fitness, as shown by the absence of significant fitness differences across quantile groups (Fig. 3b) or across the full range of BLUPs variation (Fig. 3a). This suggests that in low-quality environments, the fitness benefits of multiple paternity are associated with a broader range of consistent individual tendencies or strategies. Comparatively, except for salamanders, there has so far been no link between multiple paternity and increased offspring production for most amphibians and reptiles too [85]. One possible explanation is that in poorer environments, male and female behaviours interact more strongly to determine reproductive success: some females (perhaps those of highest quality) can afford to reproduce successfully without male care, while others may rely more heavily on multiple paternity as a strategy to mitigate infanticide risk. Importantly, the markedly lower among-individual variance in multiple paternity under high-quality food conditions suggests that females were more plastic/flexible, behaviourally, when ecological constraints were better. This is in line with previous work on plasticity in reproductive behaviour [51] and with the idea that differences in behaviour can be shaped by differences in underlying state [86, 87].

Overall, we draw three main conclusions from our results. First, in high-quality environments, all reproducing females are predicted to be similarly successful regardless of the reproductive strategy (in terms of multiple paternity) they adopt. In contrast, in the standard-quality environment, strategy seems to play a much more important role. In such conditions, some females may rely on support from males and mate multiply—possibly indicating low-quality individuals—while others might not mate multiply as they do not require male assistance to raise their offspring (male care from other aggressive or infanticidal males). If so, the incidence of multiple paternity under worse conditions may be associated with female body mass or other indicators of female quality, a relationship that may be absent in high-quality environments and warrants investigation in future studies. Alternatively, mate-guarding might be less effective in worse conditions if mating opportunities are limited, thereby allowing females to seek more males [43]. Furthermore, females in low-quality environments that do reproduce might invest heavily in a single litter at the expense of future reproduction, which could contribute to the observed pattern of fewer litters per female and higher incidence of multiple paternity within those litters, as resources are concentrated into fewer reproductive events. Second, intrinsic differences among females may explain variation in multiple paternity [21], and these differences might be more pronounced in lower-quality environments [42, 82, 83]. Indeed, high-quality resources do not limit reproduction in the same way that lower quality resources do [50]. Therefore, while some benefits of multiple paternity may be universal, others likely depend on the local environmental conditions. Third, our findings provide a valuable starting point for targeted studies aiming to disentangle the proximate and ultimate mechanisms underlying female multiple mating in mammals, particularly in relation to environmental variation within populations.

## Conclusions

Our study provides evidence that multiple paternity is beneficial predominantly in low-quality food environments in a mammalian system; and that the preliminary results of environment-specific effects of extra-pair paternity detected in birds [38–40, 42, 83, 88] might hold for polyandrous mating systems where females show multiple paternity. Similarly to how environmental heterogeneity can mediate life-history trade-offs [89–93], the strength of the adaptive value of polyandry seems to vary by environmental conditions. Whether multiple paternity is driven by active female choice [28, 79], like observed in birds, or an interaction between sexes’ traits [73] remains to be determined [94]. Importantly, though, since house mice are ecological opportunists living under diverse conditions [48, 95], our results provide general inferences about how the resource environment mediates the causes and consequences of reproductive behaviour in many species. Future studies will need to determine how individuals within populations adjust their mating decisions to changing environments by using controlled experiments too.

## Supplementary Information

Below is the link to the electronic supplementary material.

Supplementary Material 1

Supplementary Material 2

## Data Availability

The RNA sequences of the primers we used are included in the supplementary material The datasets and the R code generated and/or analysed during the current study are available in the following link: Darmis, F., & Guenther, A. (2025). Environmental quality shapes the fitness payoffs of multiple paternity. Zenodo. 10.5281/zenodo.15315050.
