# Supplementary material for: Environmental quality shapes the fitness payoffs of multiple paternity
Source: BMC Ecol Evol. 2025 Dec 1;25:134. doi: 10.1186/s12862-025-02478-5 (PMC12667171; doi:10.1186/s12862-025-02478-5)
Supplement: Supplementary file 1 — Supplementary Material 1 [file 12862_2025_2478_MOESM1_ESM.docx]

**Supplementary material for:**

**“Environmental quality shapes the fitness payoffs of multiple paternity”**

Primers used in this experiment (originate from Teschke *et al.*, 2008)

| Forward Primer | Forward Sequence | Reverse Primer | Reverse Sequence |
| --- | --- | --- | --- |
| Chr01_23F | gctgcaagaagctcacggctac | Chr01_23R | agtgtcacgtcatccaggccag |
| Chr01_25F | tcctgaacagaggactctgacc | Chr01_25R | gctagactgatactttccgacttag |
| Chr02_01F | agggctctctgacctgtagcag | Chr02_01R | tgagttcaagtccacactggtc |
| Chr02_02F | gttaaggctgccgccatcagag | Chr02_02R | agcctgacaacctggaacccac |
| Chr3_24F | tcccatgcctctaacaacctgg | Chr3_24R | acgtcttgataggcatctgtgc |
| Chr05_45F | tctggcctgtgtgcacctagtc | Chr05_45R | acaatgcttggtgactgtggtg |
| Chr08_11F | tggaacactcaagcaattccca | Chr08_11R | tagctcccaaggacagttgacg |
| Chr09_20F | tgcaggaagactcccggacttg | Chr09_20R | agactccacttgggacaactgc |
| Chr10_45F | gggagacatggtatgtctgcac | Chr10_45R | tacagtgcttctccgactaagg |
| Chr12_05F | gctctctcaaatcgatggtctc | Chr12_05R | ctttcatggtaccagaaggctc |
| Chr13_22F | tagctgatgccaagaccagtcc | Chr13_22R | agtctctccagacagcactacc |
| Chr14_16F | actgccattactgctgacatgg | Chr14_16R | gacatcggtgtgagccatctgc |
| Chr16_21F | actcatgatcacacatgctctg | Chr16_21R | taagaggccacccagctacctg |
| Chr17_09F | aggctcattctagacagactcc | Chr17_09R | acaagggtcggctgtgtgtctg |
| Chr19_08F | tcagaggcagagtctcgctagg | Chr19_08R | agttgtggctccgcccggtgtc |
| X_14F | agcaagacaggatagcacaacg | X_14R | atctcagcacctgtcccaagtg |
| X_63F | gcacactcatcaaaccacagac | X_63R | agagaaactggtgtcatcctgc |

**Table S1.** Results of model 1 testing hypotheses 1 & 2. The estimate (Log-Odds) is considered significant (highlighted in bold here) if the 95% credible interval (CI 95%) does not contain 0.

|  | **Probability of multiple paternity per reproductive event** | |
| --- | --- | --- |
| *Predictors* | *Log-Odds* | *CI (95%)* |
| Intercept | -2.16 | -7.63 – 0.37 |
| Litter size | 0.18 | -0.12 – 0.72 |
| Age | 0.25 | -0.36 – 1.27 |
| Food [standard-quality] | -2.14 | -6.78 – 1.25 |
| **Population size** | **0.83** | **0.03 – 2.32** |
| **OSR** | **0.69** | **0.02 – 1.87** |
| Age*Food [standard-quality] | 0.19 | -0.64 – 1.10 |
| OSR*Food [standard-quality]  Litter size*Food [standard-quality] | 1.15  0.25 | -1.72 – 4.25  -0.22 – 0.83 |
| OSR*Food [standard-quality] | -0.62 | -1.82 – 0.34 |
| Population size*Food [standard-quality] | -0.81 | -2.66 – 0.37 |
| Observations | 255 litters from 202 females | |

**Table S2.** Results of model 2, testing hypothesis 3a: if a multiply-sired litter is bigger than a non-multiply-sired one. The estimate (log-odds) is considered significant (highlighted in bold here) if the 95% credible interval (CI 95%) does not contain 0.

|  | **Litter size** | |
| --- | --- | --- |
| *Predictors* | *Log-Mean* | *CI (95%)* |
| **Intercept** | **1.34** | **1.12 – 1.55** |
| Probability the litter is multiply sired | 0.03 | -0.15 – 0.21 |
| **Food [standard-quality]** | **-0.22** | **-0.40 – -0.04** |
| Probability the litter is multiply sired*  Food [standard-quality]  Age | 0.18  0.01 | -0.08 – 0.44  -0.05 – 0.07 |
| Population size | 0.04 | -0.05 – 0.14 |
| Population size*Food [standard-quality] | -0.13 | -0.29 – 0.02 |
| Observations | 255 litters from 202 females | |

**Table S3.** The results of the *post-hoc* analysis of model 2 comparing the incidence of multiple paternity (MP) between and within food qualities (HQ = high-quality food; SQ = standard-quality food). The contrasts show what is compared and the estimate shows the difference between the first and the second contrasting category. The lower and upper highest posterior density (HPD; interval probability set at 0.95) credible interval are also shown, and all results are given on the log (not the response) scale. The estimate is statistically significant (bold here) if the interval does not contain 0.

| Contrast | Estimate | Lower HPD | Upper HPD |
| --- | --- | --- | --- |
| No MP in HQ *versus* MP in HQ | -0.03 | -0.21 | 0.15 |
| No MP in HQ *versus* No MP in SQ | 0.17 | -0.01 | 0.34 |
| No MP in HQ *versus* MP in SQ | -0.04 | -0.23 | 0.15 |
| MP in HQ *versus* No MP in SQ | **0.21** | **0.13** | **0.39** |
| MP in HQ *versus* MP in SQ | -0.01 | -0.22 | 0.20 |
| No MP in SQ *versus* MP in SQ | **-0.21** | **-0.40** | **-0.02** |

**Table S4.** WAIC (the Bayesian analog of AIC; Watanabe, S. (2010)**.** *Asymptotic Equivalence of Bayes Cross Validation and Widely Applicable Information Criterion in Singular Learning Theory.* Journal of Machine Learning Research, 11, 3571–3594) comparing if a model assuming a linear relationship of the log of the expected counts (Poisson model) or a non-linear relationship (but again a Poisson residual distribution) between Fitness and BLUPs is a better fit for the data. Lower (more negative) elpd_diff values indicate worse predictive performance relative to the best model. The GAMs clearly outperform the generalised linear models for both HQ and SQ.

| Dataset | Model | elpd_diff | se_diff |
| --- | --- | --- | --- |
| **HQ** | gam_model_HQ | 0.0 | 0.0 |
|  | generalised linear_model_HQ | –22.1 | 9.6 |
| **SQ** | gam_model_SQ | 0.0 | 0.0 |
|  | generalised  linear_model_SQ | –49.6 | 9.0 |


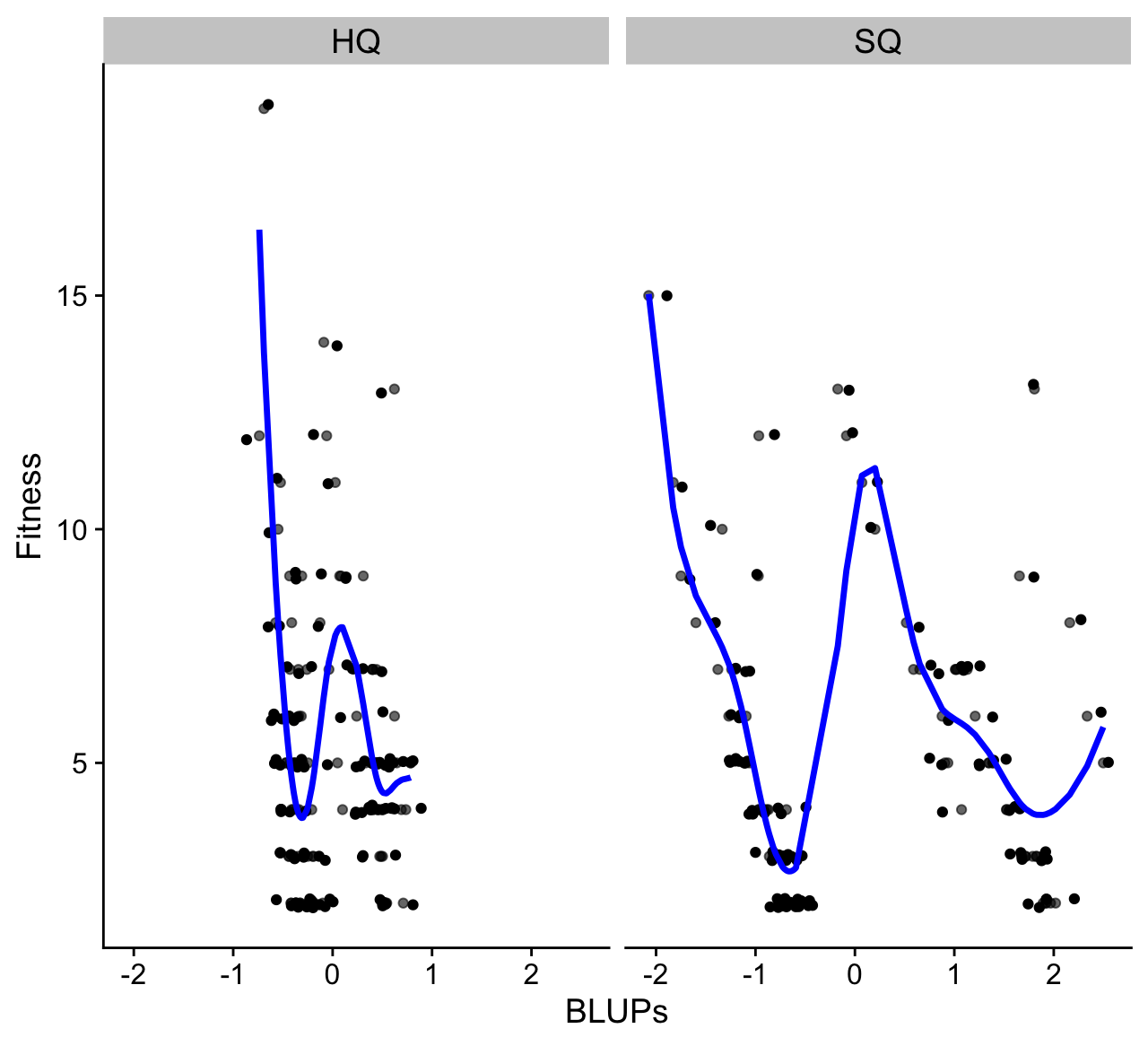


Figure S1. The relationship between fitness and BLUPs per food quality is clearly non-linear. The blue line is the shape of a GAM that smooths the line according to the data. See also table S4 above.
